# Spatial and genetic clustering of *Plasmodium falciparum* and *Plasmodium vivax* infections in a low-transmission area of Ethiopia

**DOI:** 10.1038/s41598-020-77031-z

**Published:** 2020-11-17

**Authors:** Sofonias K. Tessema, Mulualem Belachew, Cristian Koepfli, Kjerstin Lanke, Tiffany Huwe, Wakweya Chali, Girma Shumie, Elias F. Mekuria, Chris Drakeley, Endalamaw Gadisa, Bryan Greenhouse, Teun Bousema, Fitsum G. Tadesse

**Affiliations:** 1 EPPIcenter Research Program, Division of HIV, ID, and Global Medicine, Department of Medicine, University of California, San Francisco, CA USA; 2 Armauer Hansen Research Institute, PO Box 1005, Addis Ababa, Ethiopia; 3 Department of Biological Sciences, Eck Institute for Global Health, University of Notre Dame, Notre Dame, IN 465556 USA; 4 Department of Medical Microbiology, Radboud University Medical Center, 6500 HB Nijmegen, The Netherlands; 5 Faculty of Public Health, Jimma University, Jimma, Ethiopia; 6 Department of Immunology and Infection, London School of Hygiene & Tropical Medicine, London, WC1E 7HT UK; 7 Chan Zuckerberg Biohub, San Francisco, CA 94158 USA; 8 Institute of Biotechnology, Addis Ababa University, PO Box 1176, Addis Ababa, Ethiopia

**Keywords:** Parasite biology, Parasite genetics

## Abstract

The distribution of malaria infections is heterogeneous in space and time, especially in low transmission settings. Understanding this clustering may allow identification and targeting of pockets of transmission. In Adama district, Ethiopia, *Plasmodium falciparum* and *P. vivax* malaria patients and controls were examined, together with household members and immediate neighbors. Rapid diagnostic test and quantitative PCR (qPCR) were used for the detection of infections that were genetically characterized by a panel of microsatellite loci for *P. falciparum* (26) and *P. vivax* (11)*,* respectively. Individuals living in households of clinical *P. falciparum* patients were more likely to have qPCR detected *P. falciparum* infections (22.0%, 9/41) compared to individuals in control households (8.7%, 37/426; odds ratio, 2.9; 95% confidence interval, 1.3–6.4; *P* = .007). Genetically related *P. falciparum*, but not *P. vivax* infections showed strong clustering within households. Genotyping revealed a marked temporal cluster of *P. falciparum* infections, almost exclusively comprised of clinical cases. These findings uncover previously unappreciated transmission dynamics and support a rational approach to reactive case detection strategies for *P. falciparum* in Ethiopia.

## Introduction

Recent success in malaria control in endemic countries has re-ignited elimination efforts^1^. Transition from control to elimination requires improved understanding of transmission patterns within communities^2^ to better target the remaining infections that sustain or seed transmission. In low endemic settings, malaria infections are often characterized by low parasite density, commonly below the detection limit of rapid diagnostic tests (RDT) and microscopy^3,4^. These low-density infections are typically asymptomatic and may contribute substantially to onwards transmission to mosquitoes^5^.

As malaria transmission intensity declines, the distribution of malaria infections becomes increasingly heterogeneous, and infections may cluster within villages or households^6–8^. If this clustering reflects pockets of active transmission, finding and targeting these clustered infections may accelerate targeted elimination efforts^9^. Based on this assumption, individuals living in close proximity to passively detected cases are screened and treated in reactive case detection (RCD) strategies in low transmission settings^8^. The optimal and logistically feasible population that needs to be targeted in RCD remains unclear^10,11^. Several studies have attempted to determine whether geographical clusters of infections show evidence of increased genetic relatedness and thus truly reflect related transmission events^12–18^. Few studies have coupled household-level case clustering studies with information on the genetic relatedness of infections. Such studies can provide a better understanding of fine-scale transmission patterns and estimate the contribution to transmission of infections that are not detected by routine interventions.

In this study, the prevalence and distribution of asymptomatic *P. falciparum* and *P. vivax* infections surrounding passively detected RDT-confirmed clinical malaria patients was assessed using RDT and quantitative PCR (qPCR) in Batu Degaga, Ethiopia. Spatial and temporal relatedness and clustering of infections within and between households of passively detected malaria infections and controls were investigated by genotyping 26 and 11 microsatellite loci for *P. falciparum* and *P. vivax,* respectively, which had been shown previously to be useful to describe population structure^12,19–25^.

## Results

### Prevalence and clustering of malaria infections

**Figure 1:**
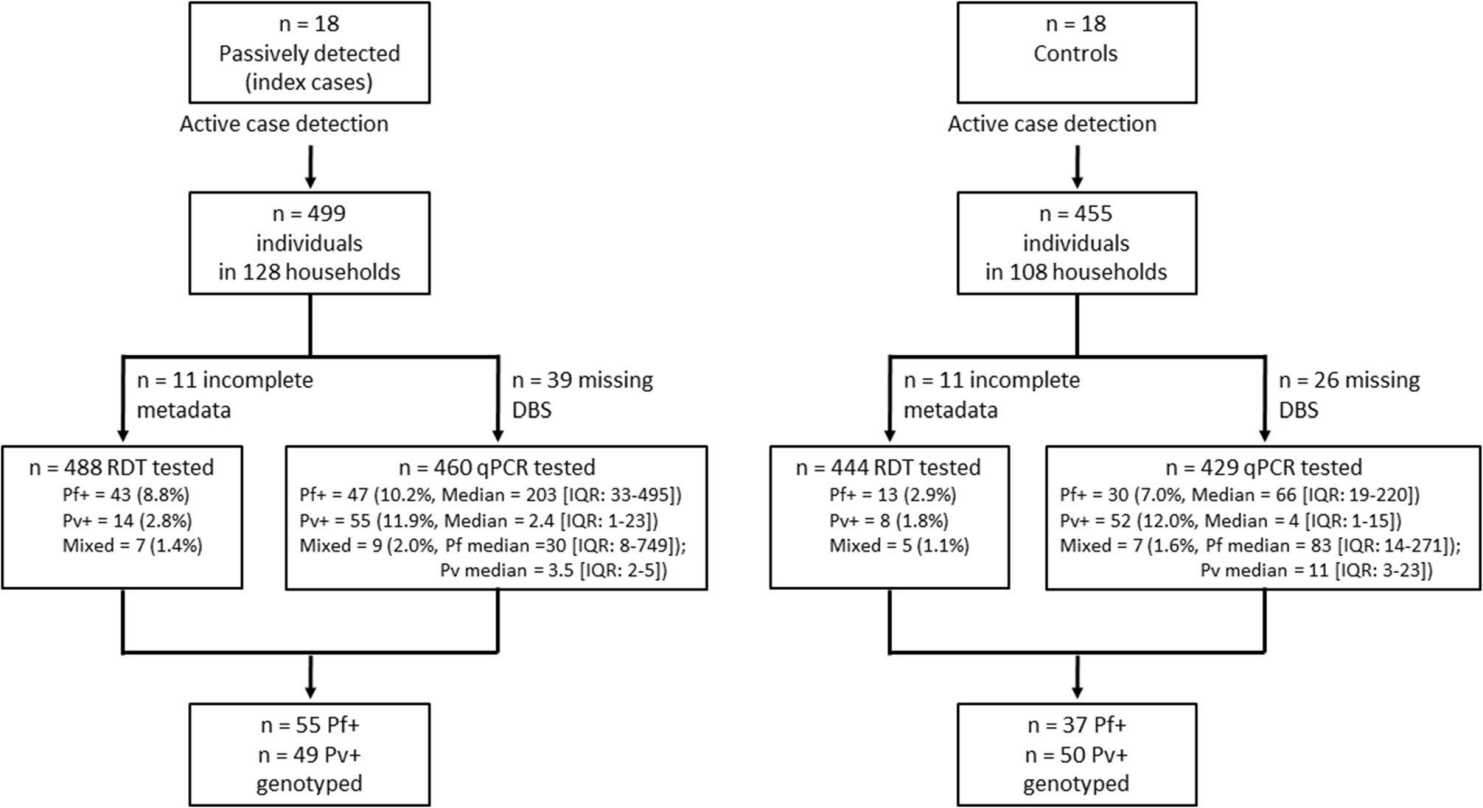
Flow chart of samples collected and genotyped. Indicated in the figure is the number of index cases and controls and individuals screened around them. Parasite identification results by RDT and qPCR are also indicated. Of all samples with complete metadata that were tested using both RDT and qPCR (n = 889) only 5 *P. falciparum* and 1 mixed species infection were qPCR negative whilst being positive. All RDT positive samples were genotyped except these six samples. Parasite densities (18S copies/µL) are indicated for each species in (median, interquartile range [IQR]). Indicated at the bottom are number of samples that were genotyped for each species. DBS, dried blood spot; RDT, rapid diagnostic test; *Pf*, *P. falciparum*; *Pv*, *P. vivax*; qPCR, quantitative polymerase chain reaction; IQR, interquartile range.

**Figure 2:**
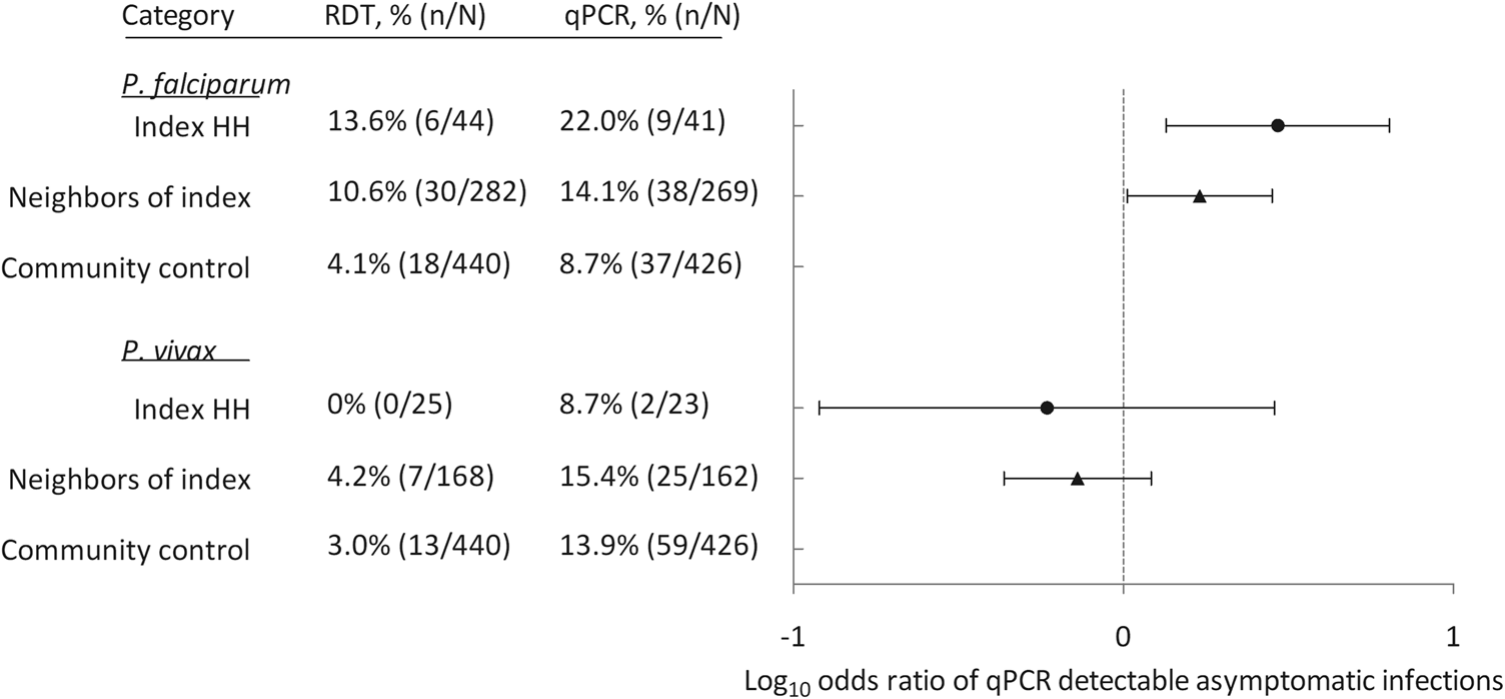
Comparison of the odds of detecting infections around households. The forest plot shows the odds ratio and 95% confidence interval for detecting asymptomatic malaria infections by 18S based qPCR in households of index cases (Index HH) and neighbors of index cases compared to control households (community control) for *P. falciparum* and *P. vivax* infections separately after excluding the index cases. Indicated on the X-axis the Log_10_ transformed odds ratio and on Y-axis are the different comparison groups. The prevalence of RDT and qPCR detectable asymptomatic infections for each household category and species are also indicted for each group to the left of the figure.

Family members and neighbors of 18 passively detected index cases with RDT confirmed *P. falciparum* (n = 10), *P. vivax* (n = 6) or mixed species infections (n = 2) and 18 controls without malaria infections participated in this study. In total, 499 individuals from 128 households around index cases and 454 individuals from 108 households around controls were successfully sampled (Fig. 1). The median number of individuals examined was 28 around index cases (interquartile range [IQR], 24–31) and 26 around controls (IQR, 22–30), with a median of 4 individuals (IQR, 3–5) examined per household. Reported history of malaria in the last 3 weeks was substantially higher among individuals in index HHs (38.8%, 31/88; *P* < 0.001) compared with their neighbors (22.2%, 92/414) and those from and around control HHs (4.3%, 19/447). In the screened population surrounding index cases and controls (excluding the index cases), prevalence of infection was 7.9% (72/913) by RDT and 21.6% by qPCR (189/877). At least one infected individual was found in 28% (66/236) and 48% (114/236; *P* < 0.001) of households by RDT and qPCR, respectively. *P. falciparum* infections detected in households of neighbors of index cases had higher parasite density (median parasite density by qPCR, 203 copies/μL; IQR, 33–576) than infections detected in households of neighbors of controls (median, 75 copies/μL; IQR, 17–209; *P* = 0.037); whilst such difference was not detected in *P. vivax* infections (*P* = 0.27). Fewer *P. falciparum* infections were detected by qPCR in households of controls (8.6%, 37/432) compared to index case households (20.3%, 12/59; OR, 2.7; 95% CI 1.3–5.6; *P* = 0.01) after excluding index cases. There was no evidence for differences in parasite prevalence by PCR between households of control and neighbors of index cases (10.4%, 41/395; OR 1.2; 95% CI 0.8–2.0; *P* = 0.37; Fig. 2). For *P. vivax* infections, no such differences were detected in qPCR parasite prevalence in relation to proximity to the household of the index case (*P* = 0.53; Fig. 2).

### Genetic complexity and diversity of *P. falciparum* and *P. vivax* infections

**Figure 3:**
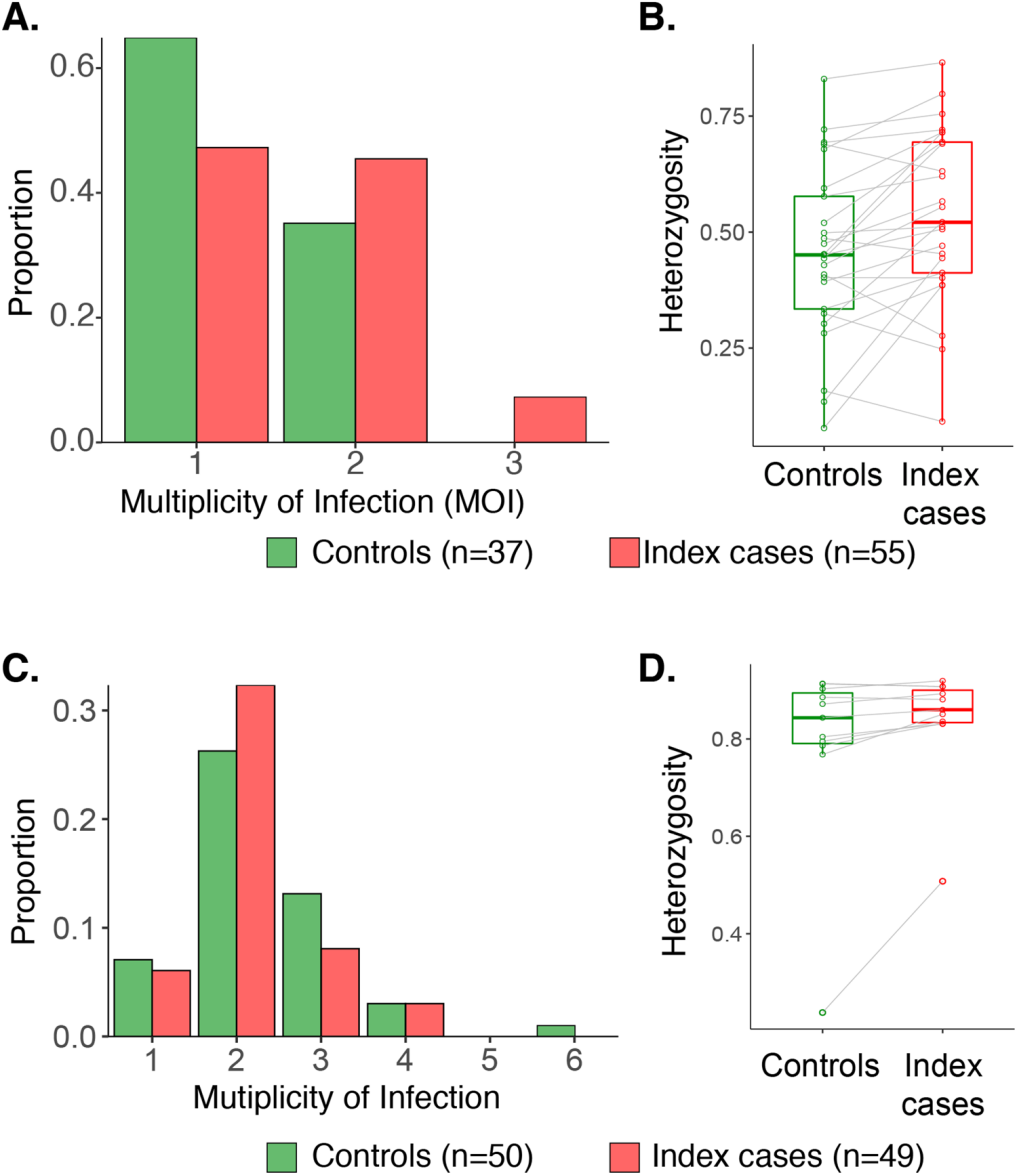
Genetic complexity and diversity of *P. falciparum* and *P. vivax* infections around index cases and controls. Distributions of multiplicity of infection (**A** and **C**) and the expected heterozygosity (**B** and **D**) of markers from samples collected in and neighboring households of index cases and controls are indicated for *P. falciparum* (**A** and **B**) and *P. vivax* (**C** and **D**). In (**B** and **D**), each dot represents one microsatellite marker. Boxplots indicate the medians and interquartile ranges of the heterozygosity of 25 loci for *P. falciparum* and 11 loci for *P. vivax*. The *P. falciparum* AS1 locus was excluded from the analyses due to the lack of diversity in this population. The whiskers indicate the highest and lowest values.

A total of 92 *P. falciparum* infections and 99 *P. vivax* infections were successfully genotyped. The number of genetically distinct parasite clones (i.e. multiplicity of infections, MOI) and population level genetic diversity were compared for infections around index and control cases for each species. In *P. falciparum* infections, the mean MOI was 1.5 (range: 1–3) and infections detected in and around households of index cases had higher MOI compared to those detected in and around control households (mean MOI: 1.6 vs. 1.4; *P* = 0.04; Fig. 3A). When stratified by index and controls, infections detected around index cases showed moderately higher genetic diversity compared to those detected around controls in *P. falciparum* (Mean H_E_: 0.56 vs. 0.49; *P* = 0.03; Fig. 3B).

In contrast to the sympatric *P. falciparum* populations, within-host diversity was consistently higher in *P. vivax*, the mean MOI was higher in *P. vivax* (mean MOI = 2.2; range: 1–6), with no difference between index and control cases (mean MOI 2.2 vs. 2.3; *P* = 0.35; Fig. 3C). The overall genetic diversity of *P. vivax* infections was high (Mean H_E_ ± SE: 0.82 ± 0.03). Similar to *P. falciparum,* infections detected around index cases showed moderately higher genetic diversity compared to those detected around controls (Mean H_E_: 0.84 vs. 0.79; *P* = 0.08; Fig. 3D).

### Spatial and temporal relatedness of *P. falciparum* and *P. vivax* infections

**Figure 4:**
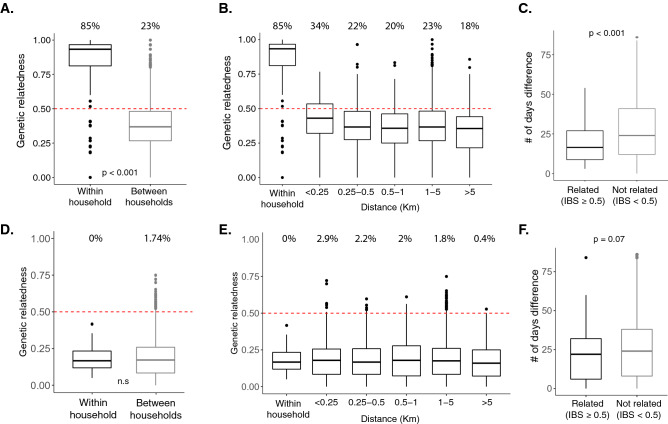
Spatial and temporal genetic relatedness of *P. falciparum* and *P. vivax* infections. Pairwise genetic relatedness is shown for sample pairs collected within and between households for *P. falciparum* (**A**) and *P. vivax* (**D**). The proportion of comparisons in each category that are related (above the 0.5 genetic relatedness threshold demarcated by the red dashed line) is shown at the top. Pairwise genetic relatedness is shown for sample pairs by physical distance bin for *P. falciparum* (**B**) and *P. vivax* (**E**). Box plots indicating the distributions of elapsed days for genetically related (IBS > 0.5) and unrelated infections are shown for *P. falciparum* (**C**) and *P. vivax* (**F**). Boxplots indicate the median and interquartile ranges, and the whiskers indicate the highest and lowest values.

Pairwise genetic relatedness in 70 *P. falciparum* samples from 57 households (with allele calls from at least 15 loci) and 91 *P. vivax* samples from 79 households (with allele calls from at least 6 loci) were compared using the IBS metric. These data were used to determine how genetic relatedness of infections varied in space and time. *P. falciparum* infections sampled from the same household were more strongly related than infections from different households (median genetic relatedness: 0.81 vs. 0.37, *P* < 0.001; Fig. 4A). The degree of relatedness did not differ depending on household distance. *P. falciparum* infections that were genetically related (IBS > 0.5) and sampled from different households (Fig. 4B), were sampled within a shorter time frame compared to unrelated infections (median number of days: 17 vs. 25, *P* < 0.001; Fig. 4C), suggesting temporal clustering of genetically related *P. falciparum* infections. In contrast, overall pairwise genetic relatedness between *P. vivax* infections was low, with no spatial (Fig. 4D,E) or temporal clustering (Fig. 4F).

### Fine-scale population structure of *P. falciparum* but not *P. vivax* infections

**Figure 5:**
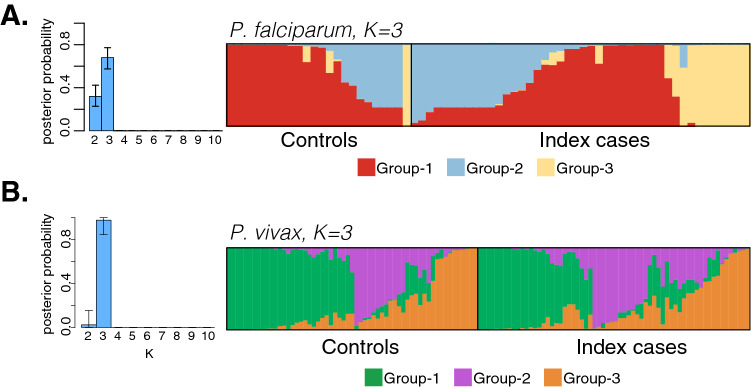
Fine-scale population structure and focal transmission of genetically related infections. Population cluster analysis of *P. falciparum* (**A**) and *P. vivax* (**B**) microsatellite haplotypes from dominant alleles for cases detected around index cases and controls. Individual ancestry coefficients and optimum K value (K = 3) are shown as inferred by MavericK^70^. Each vertical bar represents an individual haplotype, and its membership to the three population groups is defined by the different colors. Black borders separate individuals detected around controls and index cases.

Analysis of dominant alleles at each locus using MavericK identified three sub-populations (K = 3), with the highest model evidence for both *P. falciparum* and *P. vivax*. Most parasites had their origin clearly assigned to a single cluster; few of them were admixed as shown by bars partitioned into K colored segments (Fig. 5). *P. falciparum* infections detected around index cases vs. controls were comprised of different populations (Fig. 5A), with infections belonging to group-3 almost exclusively detected in and around index cases households (23% vs. 4%, *P* = 0.010) and group-1 overrepresented in controls (72% vs. 42%, *P* = 0.003). Infections assigned to group-3 were identified in five independent RCD investigations and included one individual in the control group whose infection was genetically related to one of the index cases (IBS = 0.7). Analyses of the elapsed time between samples in each group showed that infections assigned to group-3 were clustered temporally (median number of days = 16, IQR: 0–30) compared to group-1 (23, IQR: 12–42; *P* = 0.007) and group-2 (25, IQR: 7–41; *P* = 0.004). These observations support the hypothesis that infections identified through investigation around index cases are linked possibly due to recent, local spread of group-3 haplotypes during the study period. No *P. vivax* genetic groups were overrepresented either to the control or index cases (Fig. 5B).

## Discussion

In the current study, we examined spatial clustering of *Plasmodium* infections in a low endemic area in Ethiopia with a focus on asymptomatic infections in the vicinity of passively detected clinical index cases. Asymptomatic *P. falciparum* infections were clustered in index case households whilst there was no such evidence for clustering of *P. vivax* infections. *P. vivax* infections were more genetically complex and diverse than *P. falciparum* infections, with no detectable spatial or temporal clustering. We identified fine-scale focal transmission and a genetic cluster around index cases that may represent a clonal *P. falciparum* expansion, highlighting the added value of genotyping to understand the contrasting transmission epidemiology of the two *Plasmodium* species.

Spatial and temporal heterogeneity in the occurrence of infections is widely acknowledged in malaria^26,27^ and other infectious diseases^28–30^. Several countries have adopted RCD approaches that take advantage of infection clustering and assume that transmission chains, or at least transmission sources, can be interrupted by targeting infected household members. In some settings where symptomatic and asymptomatic malaria cases do not overlap spatially, clusters of infections can be missed by RCD^31^. This highlights the need for detailed studies on *Plasmodium* transmission patterns in settings aiming for elimination^32^. In the current study, we moved beyond spatial patterns in infection prevalence and examined small-scale transmission patterns by genotyping microsatellite markers. The overall genetic diversity (i.e. the mean expected heterozygosity) in *P. falciparum* infections was lower (Mean H_E_ ± SE: 0.47 ± 0.03) than previously observed in other low transmission areas of Eswatini, Zambia, and Namibia (Mean H_E_: 0.71–0.79)^12,22,33^ suggesting infrequent recombination and smaller parasite population size in the study site.

We observed a clear overlap of symptomatic and asymptomatic *P. falciparum* infections; the odds of detecting an infection within the household of clinical patients was significantly higher than in neighboring households^8,18, 34,35^. MOI and genetic diversity of infections were higher around passively detected *P. falciparum* compared to controls, indicative of higher transmission. We observed that the very strong household-level clustering for *P. falciparum* was matched with strong genetic relatedness of infections within a household. Density of asymptomatic *P. falciparum* infections surrounding clinical cases was higher than in infections detected in control households. This might indicate that the former infections were acquired more recently compared to older, very low density infections detected in controls. All of these findings corroborate that RCD is useful to detect clusters of recently acquired *P. falciparum* infections. Results from control households, however, show that a substantial number of infections is not linked to index cases. As found in other studies^36,37^, RCD alone is thus unlikely to achieve elimination.

We detected a genetic cluster of *P. falciparum* infections, present in multiple households, that was overrepresented in and around index cases. These findings suggest a possible population expansion of a single parasite genotype. We can hypothesize that possible expansion, occurring against the background of wide-spread asymptomatic infections, could indicate introduction of a clone from another site. Its association with clinical cases suggests that some infections disproportionally give rise to symptoms^18^.

As in other sites where *P. falciparum* and *P. vivax* are co-endemic, *P. vivax* was more diverse, showed high MOI, and exhibited lower levels of population structure^38–41^. *P. vivax* diversity was similar to previous studies from Madagascar (Mean H_E_: 0.80), PNG highlands (Mean H_E_: 0.82) and lowlands (Mean H_E_: 0.80) and higher than South Korea (Mean H_E_: 0.56)^42^. There was no significant difference in the odds to detect a asymptomatic *P. vivax* infection in the household or neighbors of an index case compared to a control household. There was no difference in the MOI and heterogeneity of infections around passively detected *P. vivax* infections and controls. No spatial clustering was observed for *P. vivax.* The loss of relatedness and household level clustering may reflect differences in the biology of these two *Plasmodium* species where the majority of clinical *P. vivax* cases are attributable to relapsing episodes and do not necessarily reflect an active circulating infection that involves mosquito bites^43,44^. This would contribute to considerable variation in the time between the original inoculation by mosquito bite and parasite detection in the bloodstream, and thus diffuse any genomic signal. In summary, RCD is not more effective than random screening to identify asymptomatic *P. vivax* infections in the study site.

Our findings highlight the value of genotyping approaches to infer spatial and temporal clustering of *P. falciparum* infections. Whilst infections are prevalent outside households of index cases, and thus may be missed by RCD approaches, a disproportionate number of infections may be targeted by RCD^45^. Our finding of a possible population expansion of a single parasite genotype arising from a clone that was frequently detected in clinical cases further supports RCD approaches that may interrupt clinically relevant transmission chains. The lack of spatial (genetic) clustering in *P. vivax* may be attributable to the limitations of the methods used in this study, in addition to its unique biology discussed above, calling for more robust approaches. The biology of *P. vivax*; existence of hypnozoites^43,46,47^, generation of gametocytes at the very early stage^48^, lower parasite densities^49,50^, and higher vectoral capacity^51–53^, makes it a harder species to control and one that may require a different strategy to that of *P. falciparum*^54,55^. Future studies may benefit from more sample size and including genotyping of mosquitoes paired with the human sampling in study households^56^. In summary, for *P. vivax* RCD may not be more efficient than untargeted screening to identify asymptomatic infections in the study site. Control strategies against *P. vivax* need to target hypnozoites using radical cure, as the majority of infections detected are attributable to relapsing episodes rather than active infections^57^. Justified by the very low prevalence of glucose-6-phosphate dehydrogenase deficiency^58–64^. Ethiopia is currently implementing a 14-day dose primaquine for radical cure in *P. vivax*^65–67^.

## Methods

### Study site, population and ethics statement

**Figure 6:**
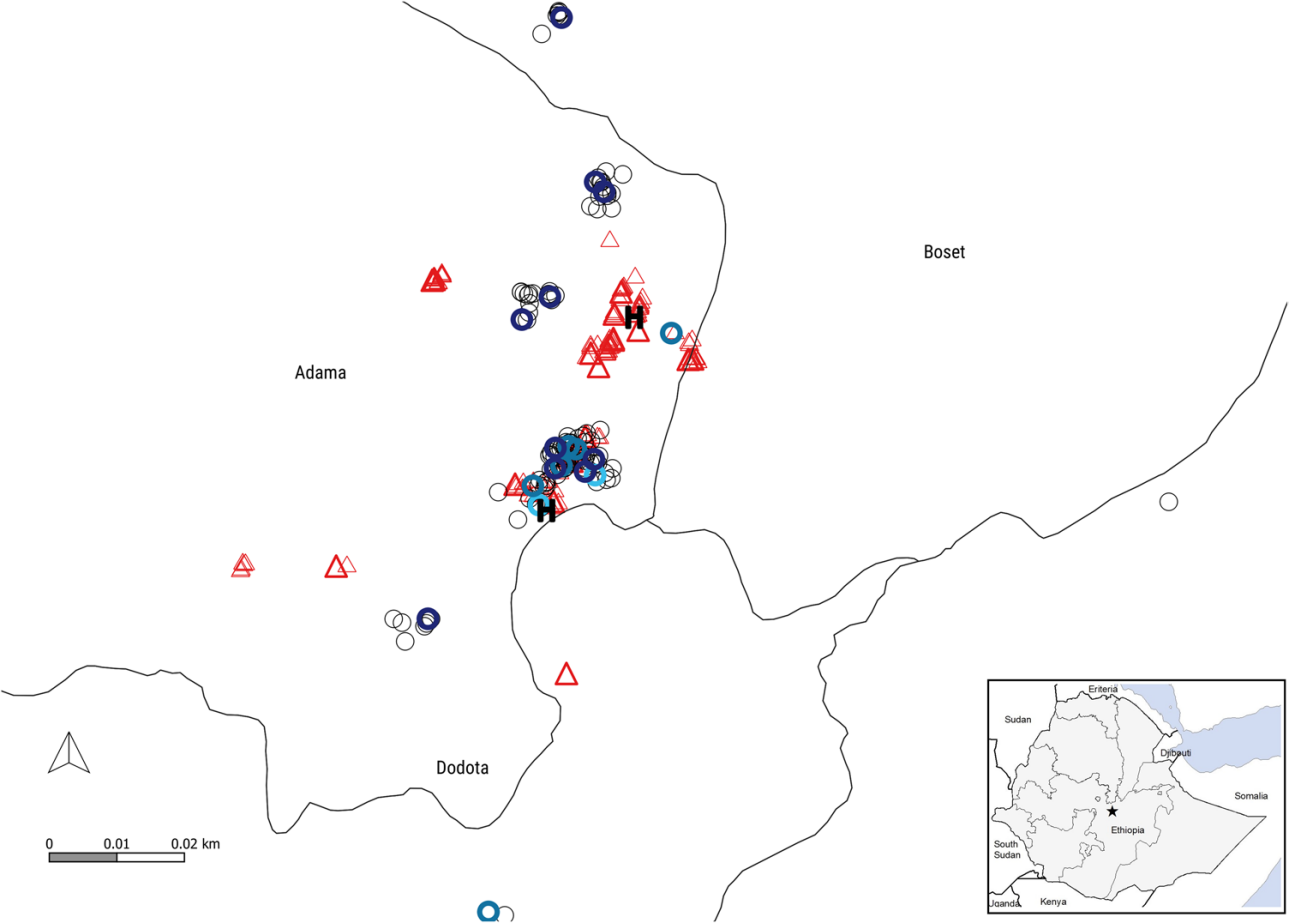
Map of study area and distribution of study households. Shown in the right bottom corner is a map of Ethiopia with boundaries indicating administrative regions (asterisk indicating the study district, Adama, Oromia region). The location of index households (thick circles; *P. falciparum*, dark blue; *P. vivax*, blue; mixed species, light blue) and neighbors of index households (thin circles) surveyed during the study are indicated together with control households (thick triangles) and neighbor households of controls (think triangles). The two health posts are indicated in black and bold letter ‘H’. The entire study area was 0.031 km^2^. The QGIS software version 3.14.16 (QGIS developer team, Open Source Geospatial Foundation Project) was used to map study households (https://issues.qgis.org/projects/qgis/). Coordinates of study household were geo-located with a handheld GPS receiver (GPSMAP 62 s; Garmin International).

This study was conducted between October and December 2016 in Batu Degaga within Adama district, Ethiopia. Adama district is located at an altitude range of 1400–2300 m above sea level, and has an estimated population of 183,502 within 38,230 households (District Health Office Report). *P. falciparum* and *P. vivax* are co-endemic in the district^5,68^ with 60% of the infections attributed to *P. vivax*^5^. The area is characterized by unstable seasonal transmission that peaks following the two rainy seasons: September to November (major) and April to May (minor). Two health posts, the lowest level governmental heath facilities, serve the study area. Only one of the two health posts (the southern health post, Fig. 6) was providing service during the entire study period. The northern health post was opened only for 3 days during the study period.

The study was approved by the ethics review boards of the Department of Medical Biochemistry at Addis Ababa University (DRERC 19/16), Armauer Hansen Research Institute (PO52/14), the National Research Ethics Review Committee (310/109/2016), and the London School of Hygiene and Tropical Medicine (10628). Written informed consent was obtained from all participants and/or parents/guardians. All experiments were performed in accordance with permission obtained from participants on the consent forms, following relevant guidelines and regulations. If participant was found RDT positive while being febrile during household level sampling, he/she was referred to the nearest health post to be treated with current first-line antimalarial drugs according to the Ethiopian national malaria treatment guidelines, artemether–lumefantrine for *P. falciparum* and chloroquine for *P. vivax*.

### Sample collection and laboratory procedures

Patients with (history of) fever that presented to the two health posts and tested positive by RDT (First Response Malaria Ag. pLDH/HRP2 Combo Card Test; Premier Medical Corporation Limited, Valsad, Gujarat, India) were classified as index cases. Patients that were suspected of malaria but tested RDT negative on the same day were included as controls. Individuals who attended the health post for vaccination, trauma, pregnancy test, antenatal care, and family planning were not considered. Index cases were independent of one another (i.e. they wouldn't be found in the same cluster of households assessed for another index case). A total of 147 patients attended the clinic during the study period of whom 52 presented with main complaint of fever. Fever was confirmed in 44 of them of whom 24 were RDT positive for malaria. Patients who were referred to the nearby health center for better treatment (n = 4), were not residents of the study area (n = 2), or did not have completed information (n = 2) were excluded from the study. Controls were recruited among patients that presented to the same health post where the index case was recruited.

During the study period, 0–6 patients attended the clinic in a day and the health extension workers were available for 2–3 days a week from 9:00–12:00 a.m. The northern health post was opened occasionally and the same health extension workers were responsible and two index cases and two controls were recruited. Household members and members of the six nearest neighboring households of index cases and controls were tested for malaria by RDT within 2 days after the index or control cases were identified^8^ and had their household geo-located with a handheld GPS receiver (GPSMAP 62 s; Garmin International). Neighboring households were selected based on proximity to the respective index case or control household upon obtaining permission from the head of the house. Socio-demographic and epidemiological data were collected, together with finger prick blood samples to diagnose malaria using RDT and to prepare dried blood spots (DBS) on 3MM Whatman papers (Whatman, Maidstone, UK) from all study participants including index cases, controls, neighbors and household members.

DNA was extracted from a 6 mm diameter punch, using MagNa Pure LC 2.0 Instrument and Total Nucleic Acid Kit—High Performance (Roche Life Sciences) with prior treatment using Buffer ATL (QIAGEN) and Proteinase K (QIAGEN)^5^. Parasites were quantified using species-specific qPCR that targeted the 18S small subunit rRNA gene^69^. qPCR positive samples were genotyped using 26 microsatellite markers for *P. falciparum* and 11 markers for *P. vivax* using previously established laboratory and data analyses protocols^12,19–23^. Mixed species infections were genotyped with both *P. falciparum* and *P. vivax* markers. Genotyping was successful in 92/93 *P. falciparum* and 99/123 *P. vivax* positive samples. For haplotype reconstruction in multiclonal infections, the predominant peak was included.

### Statistical analysis

GraphPad Prism version 5.03 for Windows (GraphPad Software, La Jolla California USA, http://www.graphpad.com); STATA 13 (StataCorp., TX, USA) and R (R Core Team, Vienna, Austria) were used for data analysis. The QGIS software version 3.14.16 (QGIS developer team, Open Source Geospatial Foundation Project) was used to map study households. Continuous variables were presented as median and interquartile range (IQR). Tests of association between two categorical variables were performed using the Chi squared test, and comparison of non-normally distributed continuous variables by Mann–Whitney U test. Odds ratios (OR) with 95% confidence intervals (95% CI) were calculated using Generalized Estimating Equations to determine the association between being parasite positive and living in households of index cases or surrounding houses. These estimates were adjusted for clustering of species-specific observations from the same household with robust standard errors and used community members not belonging to index cases or neighboring households as a reference.

Multiplicity of Infection (MOI) was defined as the highest number of alleles detected by at least two loci, reducing the impact of false positive allele calls from a single, outlier locus^12,22^. Population-level genetic diversity was determined by the expected heterozygosity (H_E_) of each locus. H_E_ was calculated for each locus using the formula ${H}_{E}=\frac{n}{n-1}[1-\sum {{p}_{i}}^{2}]$, where $n$ = the number of isolates analyzed and ${p}_{i}$ = the allele frequency of the *i*th allele in the population. Mean H_E_ was calculated by taking the average of H_E_ across all loci. To assess the genetic relatedness and clustering of *P. falciparum* and *P. vivax* infections, pairwise genetic relatedness was determined for each pair of samples using a modified identity by state (IBS) metric^22^. Briefly, pairwise IBS was calculated based on the number of shared alleles between isolates, allowing estimation of genetic relatedness from monoclonal as well as polyclonal samples. Samples were considered related if the pairwise genetic relatedness was greater than or equal to 0.5 (i.e. at least 50% of microsatellite markers shared the same alleles). This threshold reflects the degree of relatedness of meiotic siblings, which are the result of sexual recombination of different clones in the mosquito. Population structure was assessed using the program MavericK^70^, under the admixture model with 500 burn-in iterations, 5000 sampling iterations and 20 thermodynamic rungs.

### Consent for publication

This work does not contain any individual person’s data in any form (including any individual details, images or videos).

## Data Availability

The datasets used and/or analysed during the current study are available from the corresponding author on reasonable request.
